# Experimental Investigation and Performance Evaluation of Jatropha Oil-Biodiesel Blending with Kerosene for Domestic Cooking and Lighting Applications

**DOI:** 10.1155/2024/7758441

**Published:** 2024-07-22

**Authors:** Eshetu Getahun, Kefale Wagaw

**Affiliations:** ^1^Bahir Dar Energy Center, Bahir Dar Institute of Technology, Bahir Dar University, Bahir Dar, Ethiopia; ^2^Faculty of Chemical and Food Engineering, Bahir Dar Institute of Technology, Bahir Dar University, Bahir Dar, Ethiopia

## Abstract

Reducing indoor air pollution and the related costs requires developing efficient cooking and lighting technologies as well as alternative energy sources. The appropriateness of virgin jatropha (Jatropha curcas) oil, its biodiesel, and the blending of these fuels with kerosene for wick stove lighting and cooking was examined in this study. To describe the fuel performance, a range of blending ratios were examined and characterizations were made of the fuel's density, calorific value, boiling point, viscosity, and rate of fuel consumption. To assess the fuels' thermal degradation behaviors, thermogravimetric and water boiling tests were performed. An air pollution meter was used to describe the levels of indoor air pollution. According to the findings, the virgin oil from Jatropha has a kinematic viscosity of 30.1 mm^2^/s. The 50% jatropha oil and 80% biodiesel blended with kerosene showed a reduction in viscosity of 72.6% and 46.8%, respectively. The thermal efficiency of the virgin oil, its biodiesel, and blending of these fuels with kerosene was in the range of 10–48%. Complete degradation of jatropha oil, its biodiesel, and 40 : 60 ratio of jatropha biodiesel and kerosene blend was conducted in the temperature range of 480–700 k, 185–280.6 K, and 100–300 K, respectively. The activation energies of jatropha oil, biodiesel, and kerosene blend (40 : 60) were 191.3, 73, and 25 kJ/mol, respectively. The average concentrations of particulate matter and carbon monoxide for pure jatropha oil biodiesel were 209.71 *µ*g/m^3^ and 5.5 mg/kg, respectively. Thus, jatropha biodiesel and its blending with kerosene are suitable fuels for cooking and lighting operations in rural communities who are living far from the electrical grid compared with virgin oil fuel.

## 1. Introduction

Energy directly raises the standard of living for people everywhere by contributing significantly to national development. When contemporary energy is accessible and reasonably priced, it is extremely advantageous. Over 2.5 billion people rely on conventional energy to meet their needs for cooking and lighting, and 1.06 billion people worldwide lack access to modern energy [1]. Hence, access to sustainable energy, affordable, and reliable sources brings poverty reduction and economic development to every country around the world [2]. Fossil fuels, which are extremely scarce and have a major negative impact on the environment, currently dominate the energy landscape despite tremendous advancements in the development of renewable energy technologies [1, 2]. A critical first step in reducing the effects of climate change and global greenhouse gas emissions is the paradigm shift from conventional fuel sources to renewable energy systems [3].

Approximately 500 million people in sub-Saharan Africa lack access to modern energy and are instead dependent on nonsustainable sources of solid biomass fuels including firewood, animal dung, and agricultural residues [4, 5]. For their basic lighting and cooking needs, almost 80% of people in sub-Saharan Africa (SSA) rely on solid biomass. Approximately 96% of Ethiopians get their energy from traditional biomass [6]. In Ethiopia, the major fuel for cooking and baking is biomass energy.

For domestic cooking, firewood is the major fuel source in developing countries like Ethiopia, which can aggravate the deforestation process [7]. Furthermore, extensive greenhouse gas emissions and climate change are typically caused by cooking with biomass on conventional, inefficient cook stoves.

In Ethiopia, baking and cooking are the most energy-intensive processes. Firewood and charcoal are used extensively, and this leads to significant deforestation and soil erosion in the country [7]. Furthermore, biomass energy emits more greenhouse gases into the atmosphere, has lower heat contents, and burns more slowly [7, 8]. Liquid petroleum gas (LPG), ethanol, kerosene, and other fuels are being used for cooking and heating in an effort to overcome these challenges. However, in rural communities, the use of ethanol and kerosene as fuel for cooking and lighting is restricted for safety consideration, which poses a health risk. Additionally, breathing and lung cancer might be brought on by the emissions from burning kerosene [9].

Nowadays, there is a greater focus on using alternative energies to replace fossil fuels due to concerns about extreme climate change and environmental protection [10]. The limited availability and unstable cost of fossil fuel-based energy, such as kerosene, have sparked a renewed focus on developing alternative energy sources that can successfully replace kerosene in cooking operations. Petroleum fuel prices are currently soaring and becoming unstable, making them unaffordable for low-income countries like Ethiopia. Therefore, it is imperative to develop a clean, safe, and easily implementable alternative energy source for wick stoves. Vegetable-based oils are seen from this angle as reliable and substitute fuel sources, particularly in rural areas. Since plant virgin oils have the ability to emit significantly fewer greenhouse gases and improve energy security, they offer enormous promise for use as alternative fuels [11]. Nonetheless, the primary limitation during cooking processes is thought to be the high viscosity of the plant oils. Researchers have tried a number of techniques to lower the oil's viscosity. Some of these techniques include breaking up the plant oil, mixing it with alcohol, degumming, dewaxing, chemically changing the plant oils to turn them into bio-diesel through a process called alcoholysis, and heating the plant oils before using them [12, 13]. Of these techniques, the transesterification reaction method is the most effective for producing biodiesel from plant oil and animal fats. Biodiesel can be made from a variety of oilseeds, depending on availability and manufacturing capacity. Jatropha curcas, one of these oil seeds that can be grown in wastelands and semiarid environments, has the potential to make biodiesel. It is cultivable on desert or arid soils, requires less water and fertilizer, and is not suitable for cattle grazing. Depending on the weather, the oil content ranges from 30% to 40%, which is a significant amount [14, 15]. Jatropha (Jatropha curcas) is a plant in the Euphorbiaceae family that is mostly grown for its oil, which is toxic and not utilized by humans [14]. Because it is environmentally benign, renewable, and simple to manufacture in rural regions, the oil is a possible substitute for producing biodiesel. It follows that jatropha oil cannot rival human food and guarantees practical jatropha production with very little inputs. Although Jatropha plants have the potential to be a valuable source of biofuel in Ethiopia, various obstacles, including a lack of economical equipment, low knowledge among the communities, and technical limitations, have hindered the use of the oil for cooking and lighting.

The calorific value of Jatropha oil as fuel is approximately 39.65 kJ/kg, which is comparable to the calorific value of kerosene (43.50 kJ/kg). Nevertheless, Jatropha oil's high viscosity makes it impossible for it to burn efficiently during the cooking and lighting processes, making it unsuitable for use as a cooking fuel in typical wick-type local cook stoves [16]. Various sources claimed that extensive research had been conducted on the use of jatropha oil for lighting and cooking [7, 11, 12, 17, 18]. Currently, attempts are being made in Ethiopia to create cooking stoves that use pressure and gravity burners to burn plant oils as fuel [11]. However, because these cook stoves are inefficient in using the oil, alternative methods of solving the problem such as fuel blending are necessary. To the best of the authors' knowledge, therefore, not much research has been conducted in the public domain to demonstrate the suitability of blending kerosene with jatropha oil/biodiesel for use in household wick stoves. Furthermore, there is limited information about the thermodynamics and kinetics of the oil and fuel blending reaction mechanism and indoor air pollution levels.

Hypothetically, it is possible to reduce indoor air pollution and enhance the efficiency of cooking and lighting in rural communities by utilizing jatropha oil and its biodiesel and mixing these fuels with kerosene. Therefore, the objective of this study was to investigate the effect of various blends of jatropha virgin oil and biodiesel blended with kerosene on the improvement of cooking and lighting technology in Ethiopia. Detailed kinetics of combustion in addition indoor air pollution levels of the liquid fuel were also characterized. The performance was also compared with conventional cooking technologies.

## 2. Materials and Methods

### 2.1. Materials

The Jatropha curcas was collected around Dessie town, Ethiopia. Analytical grade kerosene was purchased from the supplier for blending with jatropha oil and biodiesel. Analytical grade methanol and KOH were also purchased from the supplier and used as reactants and catalysts, respectively, for the production of biodiesel. A K-type thermocouple to measure the boiling temperature, a bomb calorimeter to measure heating values, the water boiling test equipment to measure cooking efficiency, and a viscometer and hydrometer to measure viscosity and density were used for this experiment. Moreover, a thermo-gravimetric analyzer (TGA) was used to analyze the thermal and reaction kinetics behavior of the fuel. Furthermore, the degree of indoor air pollution was also measured using an emission meter.

### 2.2. Experimental Procedure

**Figure 1:**
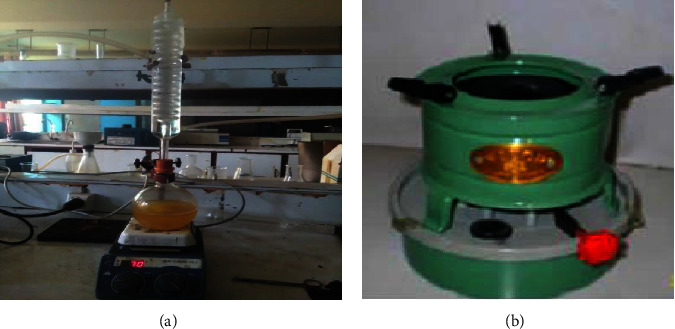
Experimental setup, (a) biodiesel production set up, and (b) local wick stove for burning the fuel.

Different ratios of jatropha oil and its biodiesel were combined with kerosene in this study. Blends of oil and biodiesel with kerosene in the volumetric ratios of 0 : 100, 5 : 95, 10 : 90, 20 : 80, 30 : 70, 50 : 50, 60 : 40, 80 : 20, and 100 : 0 were created in order to determine the possible blending ratio that could be achieved without compromising stove performance. For this experiment, a wick stove that is commonly used in the community was selected to assess the fuel blending ability (Figure 1(b)). Fuel performance and cook stoves were characterized by a number of variables, including density, viscosity, boiling point, fuel consumption rate, flashpoint, and calorific value of the fuel.

#### 2.2.1. Oil Extraction

The jatropha seeds were collected from producers around Dessie town in the Amhara region. The seeds were sun-dried for two days and then ground for suitable particle size. A mechanical press machine was utilized to extract the oil. The oil was then purified through degumming and treated with anhydrous Na_2_SO_4_ to remove any moisture. The purified oil was used to synthesis biodiesel and direct blending with kerosene.

#### 2.2.2. Biodiesel Synthesis

The biodiesel production experimental setup is displayed in Figure 1(a). Reflex condenser, chiller, and magnetic hot plate are all part of the arrangement. With a molar ratio of 1 : 6 for oil to methanol and a synthesis time of one and a half hours at 65°C, biodiesel was produced using the conventional technique through base-catalyzed transesterification [19].

#### 2.2.3. Properties of Biodiesel and Blending Fuels

The thermal performance tests, oil-kerosene mix, and biodiesel-kerosene blend were examined in accordance with accepted protocols. Standard bomb calorimeter measuring methods were used to assess the fuel content and calorific values of the blending fuels. To calculate the calorific value, a bomb calorimeter (Model: IKC200), calibrated using benzoic acid tablets, with a pressure vessel filled with 30 bar of 99% pure C-248 oxygen was utilized.

#### 2.2.4. Stove Performance Characterization

A water boiling test (WBT) calculation approach was used to assess the wick stove's performance [17].

#### 2.2.5. Thermal Efficiency

The following formula was used to get the thermal efficiency percentage:

(1) $$\begin{array}{c} \eta_{\text{th}}=\frac{\text{Mn}*\text{Cp}\left(T_{b}-T_{o}\right)+\text{Me}*L}{\text{Mf}*\text{Hv}} \end{array}$$

#### 2.2.6. Burning Rate (Rc)

This indicates how much fuel is being used at what pace while the water is boiling. This formula was used to determine it.

(2) $$\begin{array}{c} \text{Rc}=\frac{\text{Mf}}{t} \end{array}$$

#### 2.2.7. Specific Fuel Consumption (SFC)

The specific fuel consumption, which is defined as the amount of fuel needed to produce a unit output, such as cooked beans, boiling water, or loaves of bread, is typically used to calculate the fuel-saving efficiency [17]:

(3) $$\begin{array}{c} \text{SFC}=\frac{\text{Mf}}{M1} \end{array}$$

#### 2.2.8. Firepower (FPc)

The average power output (in Watts) of the stove during the cooking process is determined by calculating the ratio of the wick stove's energy consumption per unit of time.

(4) $$\begin{array}{c} \text{FPc}=\frac{\text{Mf}*\text{Cv}}{60*t} \end{array}$$

#### 2.2.9. Rate of Evaporation (Wc)

This is the rate at which water evaporates while cooking, and it is calculated as

(5) $$\begin{array}{c} \text{Wc}=\frac{M2}{t} \end{array}$$

#### 2.2.10. Thermo-Gravimetric Analysis

The experiments were performed using a thermo-gravimetric analyzer (BJHENVEN Analysis System, model, ATAT2012). To appropriately maintain pyrolysis conditions, high-purity nitrogen gas was used as the inner gas. A sample size of roughly 48 mg was employed for each fuel, and the N_2_ flow rate was 10 ml/min.

#### 2.2.11. Kinetic Analysis

Thermogravimetric data are used by the majority of the oil industry to characterize the materials and study the kinetics and thermodynamics of the reactions and transitions of oil samples. The thermal conversion of the fuels was examined using the liquid fuel kinetic analysis method. The fuel conversion rate, d*x*/d*t*, is represented as follows [20]:

(6) $$\begin{array}{c} \frac{\text{dx}}{\text{dt}}=\text{kf}\left(x\right)=k{\left(1-x\right)}^{n} \end{array}$$

where *x* is given by

(7) $$\begin{array}{c} x=\frac{\text{Wo}-\text{Wt}}{\text{Wo}-\text{Wf}} \end{array}$$

Thermogravimetric analysis indicated that the reaction is first order, *n* = 1. Thus, equation (6) becomes

(8) $$\begin{array}{c} \frac{\text{dx}}{\text{dt}}=k\left(1-x\right) \end{array}$$

If the reaction is nonisothermal, the above equation can be written as

(9) $$\begin{array}{c} \frac{\text{dx}}{\text{dT}}*\frac{\text{dT}}{\text{dt}}=k\left(1-x\right) \end{array}$$

Arrhenius indicated that the reaction rate constant k is given by

(10) $$\begin{array}{c} k=\text{Aexp}\left(\frac{-\text{Ea}}{\text{RT}}\right) \end{array}$$

Substituting equations (10) into (9) gives

(11) $$\begin{array}{c} \frac{\text{dx}}{\text{dt}}=\frac{A}{B}\;\exp\left(\frac{-\text{Ea}}{\text{RT}}\right)\left(1-x\right) \end{array}$$

The fuel consumption rate throughout the combustion process is expressed by equation (11). In this work, the activation energy was determined using nonisothermal TGA by plotting log10 (d*x*/d*t*) vs 1000/Txi for a particular conversion value, *x*, where the slope equals −Ea/R.

#### 2.2.12. Temperature Profile

**Figure 2:**
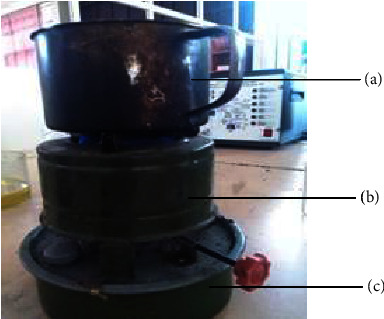
Locally used wick stove loaded with pot. (a) Water holding pot, (b) air protecting, and (c) fuel holding.

The temperature profile was determined using jatropha oil and biodiesel blended with kerosene fuel loaded onto a wick stove. About 200 g of cold clean water was weighed and poured into the aluminum pot (Figure 2). Fuel consumption and water vaporization were determined and recorded.

#### 2.2.13. Indoor Air Pollution (IAP)

Using the IAP meter (model number 1328), the pollution levels (CO and PM) were determined. The IAP meter was held on the kitchen, and an air sample can be measured every 5 minute, with the help of a sample rate switch. In order to be transferred to a computer later, the data are automatically saved on an SD memory card.

## 3. Results and Discussion

### 3.1. Properties of Jatropha Oil and Biodiesel

The distinct physicochemical characteristics of the extracted jatropha curcas oil, its biodiesel, and kerosene are shown in Table 1. The findings showed that, in comparison to kerosene, jatropha curcas oil and its biodiesel fuels had greater flash points, indicating that they are less flammable (Table 1). When it comes to handling safely and preventing fire incidents during cooking and lighting, flammability is extremely important [21]. In Ethiopia's rural areas, where communities lack sufficient knowledge about flammability, which contributes to cooking fire events, the safety of jatropha biodiesel proved crucial in managing and reducing fire incidents while cooking and lighting. In comparison to kerosene viscosity (3.1 mm^2^/s), the kinematic viscosity of the jatropha oil (Table 1) was much higher at 30.1 mm^2^/s. This suggests that the fuel has a far lower capillary effect on the movement of fuel in the wick stove and lamp during cooking and lighting. Accordingly, a pressured cook stove is an alternate stove to a conventional cook stove for using this fuel [22]. However, the cooking efficiency of pressured cook stoves is limited; therefore, fuel blending was necessary to make use of the oil in the conventional stoves that are now in use, such as wick stoves.

Jatropha oil also had a comparatively low acid content (low free fatty acid content), and the transesterification reaction was employed to directly reduce the fuel's viscosity by producing biodiesel [23]. Table 1 illustrates that the gross calorific values of biodiesel and jatropha oil fuels were 6.3% and 13.2% lower, respectively, than those of kerosene. This difference was significant for fuel optimization as it allowed for the optimal blending ratios to be obtained for cooking efficiency.

### 3.2. Properties of Blending Fuels

Tables 2 and 3 show the physicochemical properties of jatropha oil and its biodiesel mixed with kerosene, respectively. The most important characteristic of biodiesels is their viscosity, which has a significant effect on how well the fuel works during cooking and lighting. The mixed fuels' viscosities were significantly lowered, according to the results (Tables 2 and 3). Up to a 50 : 50 ratio was used to blend jatropha oil with kerosene because the fuel's performance decreased significantly below this point. The viscosity of the blended gasoline decreased as the proportion of jatropha oil in the mixture rose. Reports also showed that increasing the blends' diesel and kerosene amount decreased the viscosity and density of the vegetable oil and biodiesel [24]. In this investigation, blending 50% jatropha oil and 80% biodiesel with kerosene produced viscosity reductions of 72.6% and 46.8%, respectively. Overall, the data suggested that increasing the blending ratio of the oil and biodiesel with kerosene led to a trend of decreasing density and viscosity.

In contrast, the heating value of jatropha oil and biodiesel slightly increased as the partial substitution of kerosene increased (Tables 2 and 3). This indicates that the fuel heating value depends on the composition of the fuel. Biodiesel fuel had a calorific value that was approximately 6% lower than kerosene fuel.

### 3.3. Performance Evaluation

**Figure 3:**
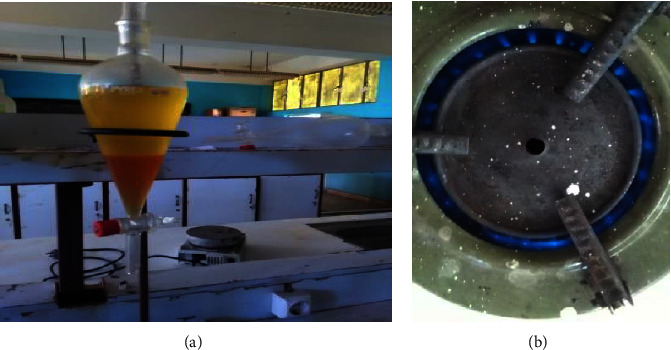
Appearance of the biodiesel fuel cooking process, (a) purified jatropha oil biodiesel at the top and glycerol at the bottom, and (b) biodiesel blue flame during cooking.

The appearance of the biodiesel separation from glycerol and the blending fuel cooking process is presented in Figures 3(a) and 3(b). In the biodiesel separation process as shown in Figure 3(a), the biodiesel was in the top and the glycerol was in the bottom.

#### 3.3.1. Water-Boiling Tests (WBT)

The viability of using kerosene-blended biodiesel and jatropha oil as possible cooking liquid fuels in the wick stoves that are currently in use in the marketplaces was assessed using water-boiling experiments.  Table 4 displays the average values of the stove performance characteristics for the blending of biodiesel with kerosene and jatropha oil. When pure jatropha oil was combined with kerosene in the existing wick cook stove and lump, the mixture showed a transparent blue flame and produced no smoke up to a 10 : 90 oil-to-kerosene mixing ratio (Figure 3(b)).

Nevertheless, ocular observation showed that the performance of the stove and lump considerably decreased when the partial substitution of jatropha oil in kerosene increased beyond 10%, and the flame length became extremely short, indicating that there was not evenly distributed heat on the cooking pot. This suggested that the viscosity of the jatropha oil is quite high, making it difficult to move the liquid fuel through the cookstove wicks and lowering the fuel's efficiency. Therefore, the best cooking performance might be achieved by blending up to 10% of jatropha oil with kerosene.

Conversely, the combination of biodiesel and jatropha oil-kerosene burned with a clear blue flame and created no smoke at any mixing ratio. Nevertheless, it was also noted that the burned fuel had a pleasant scent when the biodiesel content was reduced. This might be because kerosene has a greater ester content, which gives it a pronounced sweet scent and causes deposits of black carbon to deposit at the bottom of the cooking pot [19]. However, the sweet smell rapidly reduced along with the black debris on the pot as the biodiesel proportion increased. For the less viscous fuels, the lump wick and cook stove capillary action moved more quickly. The fuel viscosity reduced as the amount of kerosene substituted in the fuel mixture increased, which led to a short boiling time during cooking. According to Table 4, the boiling times for pure kerosene and biodiesel fuel were 11.6 and 26 minutes, respectively. The average boiling time for blending biodiesel and kerosene was 22.6 min for an equal volume of water, which was enough to cook any kind of food item.

The particular fuel consumption and firepower were impacted by the combined influence of viscosity and colorific value. As Table 4 illustrates, low fuel viscosity and high gross heating value led to low fuel usage and a quick time to boil water. Moreover, the thermal efficiency of biodiesel was 34% and pure kerosene was 60%. Blending kerosene fuel with biodiesel has an average thermal efficiency of between 33 and 60%. Pure kerosene had 1.4 kW of firepower, whereas biodiesel had 1.1 kW. For mixing biodiesel with kerosene fuel, an average firepower of 1.2 kW was obtained. According to Table 4, the blended fuels had evaporation and burning rates of 4.7–6.8 g/min and 1.5–19 g/min, respectively. The rate of burning and evaporation decreased significantly as the viscosity of the fuel increased, but the specific fuel consumption increased.

Table 4 suggested, in general, that jatropha oil and mixed fuels that contained more than 10% of jatropha oil were not appropriate for use in the cookstoves and lumps that are now in use. However, because of their good performance, jatropha oil biodiesel and its blending with kerosene were a significant and acceptable fuel in the cookstoves that were already in use, such as wick stoves and lumps for rural communities.

Despite the fact that the biodiesel blended with jatropha oil has a higher performance efficiency than pure biodiesel, given the volatility of kerosene prices at the moment, pure biodiesel itself may be used as a potential liquid fuel in the wick stoves and lumps that are currently in use. It had a low degree of indoor air pollution, as indicated by Figure 3(b), a pure long blue flame, no sweet scent, and black matter on the pot.

#### 3.3.2. Thermo-Gravimetric Analysis

In the first fuel degradation zone, the oil was started to dry, and the weight loss could be the result of water evaporating from the oil. By heating the oil, the second fuel degradation zone occurred between 300 and 480 K, indicating the loss of more volatile compounds from the oil. In the third degradation zone, the oil completely degraded between 480 and 700 K in temperature. Complete degradation of jatropha oil took place without any residual mass being left.

**Figure 4:**
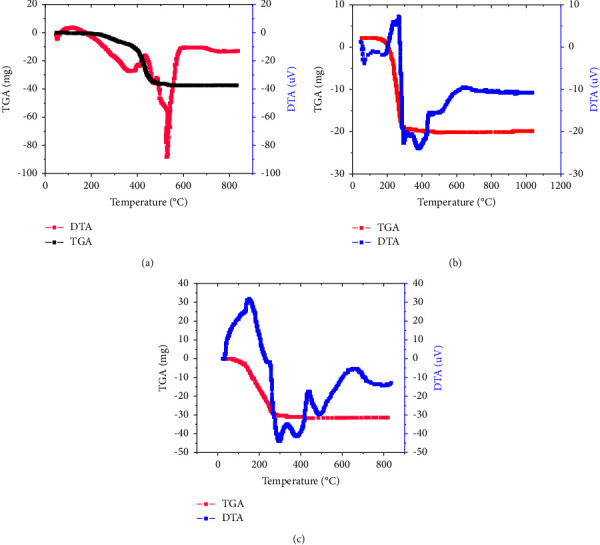
TGA and DTA analysis of (a) jatropha oil, (b) jatropha biodiesel, and (c) blending of 40% biodiesel and 60% kerosene.

In addition, the DTA curve indicated the presence of two principal peaks. The first peak, which was observed at 353 K, may have resulted from the large triglyceride molecule breaking down into smaller organic molecules. At a temperature of 523.6 K, the second peak appeared, signifying the complete breakdown of the organic molecules (Figure 4(a)). This result is consistent with a study that Biswas and Sharma described [25]. The results also showed that because jatropha oil has a high viscosity, it must evaporate at a high temperature, making it difficult to use the oil directly for cooking and lighting. This indicated that jatropha oil is an extremely heat-resistant fuel for lighting and cooking.

Figure 4(b) also shows the thermal behavior of the biodiesel made from jatropha oil. Three stages of thermal deterioration were visible in the biodiesel's TGA curves. The first involved heating the biodiesel from room temperature to 185 K, which eliminated some of the trace amounts of water that were still present. According to Jain and Sharma, the second phase thermal degradation occurred between 185 and 280.6 K, revealing the vaporization and/or pyrolysis of the methyl esters, primarily methyl linoleate and oleate [26]. Three significant mass loss peaks were seen in the jatropha biodiesel DTA curve at temperatures of 180, 292.7, and 384.1 K, respectively. These peaks indicated the removal of water, the disintegration of a big molecule, and the total degradation of the biodiesel (Figure 4(b)). It is feasible to conclude from the TGA study of the thermal behavior that the biodiesel made from jatropha oil was less thermally resistant. This suggests that the biodiesel is a very good fuel for Ethiopia's rural communities to use for lighting and direct cooking.

Figure 4(c) displays the TGA analysis and DTA curves for the thermal degradation of biodiesel made from jatropha oil combined with kerosene (40 : 60) at various heating rates. According to the TGA curve, the mass of the blended fuel began to decrease about 100 K and did so until all of the biodiesel in the sample evaporated at a temperature of 300 K. After that, the mass of the fuel stayed constant, suggesting that the fuel had completely degraded. Furthermore, three primary mass loss peaks were visible on the DTA curve. These maxima were recorded at 350 K, 400 K, and 550 K, indicating that the fuel had completely broken down by the time the temperature reached 350 K.

#### 3.3.3. Reaction Kinetic Analysis

The activation energy of the fuels was examined using the thermo-gravimetric technique. The thermo-gravimetric study yielded the first-order kinetics, which was used to calculate the activation energies of the fuels [27].

**Figure 5:**
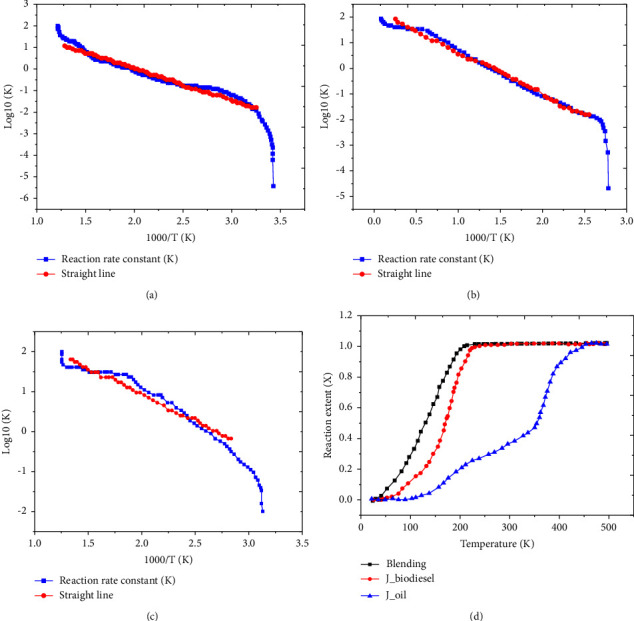
Determination of activation energy and reaction extent, (a) jatropha oil, (b) jatropha oil biodiesel, (c) 40% biodiesel with 60% kerosene fuel blend, and (d) reaction extent of fuels (X).

Figure 5 shows the activation energies and reaction lengths of biodiesel, jatropha oil, and the 40 : 60 ratio of biodiesel to kerosene. To compute kinetic parameters like Ea and A, the Arrhenius plot is crucial. For all samples, the rate constant of the reaction, log10 (K) [*K* = d*x*/d*t*], showed a linear relationship with 1/T in the result, suggesting that the fuel sample reaction was a first-order reaction. Thus, the kinetic parameter constants can be determined from the slope of the graph. The activation energies of jatropha oil, J. oil biodiesel, and 40 : 60 ratios of biodiesel with kerosene were 191.3, 73, and 25 kJ/mol, respectively (Figures 5(a), 5(b), and 5(c)). Additionally, the linear correlations (*R*^2^) of the blending fuels (40 : 60), jatropha oil, and Jatropha oil biodiesel were 0.9388, 0.938, and 0.924, respectively, indicating that the first-order kinetics can adequately describe the thermal breakdown of the fuels. This suggested that the correlation demonstrated the greater heat degradation susceptibility of blended fuels and biodiesel compared to pure jatropha oil. According to Figure 5(d), pure jatropha oil exhibits a lower reaction extent than biodiesel and its mixing fuels. It was understood that the conversion increased with temperature and that 280 k of temperature was required for full conversion. Nevertheless, the full conversion of pure jatropha oil required a temperature greater than 500 K. This indicated that jatropha oil was no longer capable of withstanding further heat deterioration, indicating that it was unsuitable fuel for use in rural communities for both direct cooking and lighting.

#### 3.3.4. Indoor Air Pollution

**Figure 6:**
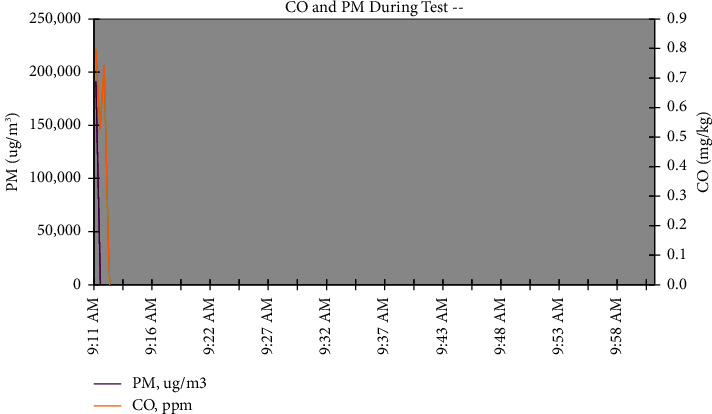
Concentration of CO and PM of jatropha oil biodiesel during cooking.

Currently, the greatest global threat to human health and the environment is air pollution. Reduced environmental air pollution could help countries combat a variety of illnesses, including heart disease, stroke, lung cancer, and acute and chronic respiratory conditions like asthma [28].  Figure 6 shows the amounts of PM and CO concentrations for the biodiesel made from jatropha oil. During the cooking process, the concentration of PM and CO for pure jatropha oil biodiesel was found to be at a low level, with an average magnitude of 209.71 *µ*g/m^3^ and 5.5 mg/kg, respectively. On the other hand, early in the cooking process, the concentrations of CO and PM rose. These could be caused by unstable air-fuel ratios, which result in incomplete fuel combustion and increased PM and CO emissions. After the cooking process reached a stable point, the levels of CO and PM were much lower and the indoor air pollution was eliminated, indicating that pure jatropha oil biodiesel, in addition to kerosene and its blends, was a good fuel source for domestic cooking and lighting.

### 3.4. Comparisons of Cookstoves

Improved cook stoves and efficient cooking fuels are crucial for lowering environmental pollution, especially indoor air pollution and illnesses linked to cooking.  Table 5 presents a comparison of various cookstoves based on their average performance metrics. When compared to wick biodiesel and its blending fuel stove, the environmental pollution produced by Lakech, Mirt, Gonze, and Tikikil stoves is lower, despite their higher thermal performances. When cooking on a wick stove, biodiesel and its blended fuel showed lower CO emissions than a kerosene burner (Table 5). This suggested that jatropha biodiesel was suitable for use in the current wick stoves since it showed significant benefits during the cooking and lighting processes when combined with kerosene fuel.

## 4. Conclusions

The appropriateness of jatropha oil and jatropha oil biodiesel and the blending of these fuels with kerosene for cooking and lighting purposes were examined in this study. The result showed that because pure jatropha oil has a high viscosity, it is not a good fuel for wick stoves used for cooking or lighting. When kerosene and jatropha oil were blended, it was observed that the high viscosity caused inefficiency in lighting and cooking. The viscosity of the 50% jatropha oil and 80% biodiesel blended with kerosene was reduced by 72.6% and 46.8%, respectively. When kerosene and jatropha oil were blended in a ratio of 10 : 90, low stove performance was observed. The thermal efficiency of the blending of biodiesel and kerosene ranged from 34 to 48%. The average firepower of mixing of kerosene and biodiesel fuel was 1.2 kW. These fuels burned at a rate of 1.5–19 g/min and evaporated at a rate of 4.7–6.8 g/min, respectively. The results of the thermogravimetric analysis showed that jatropha oil required more activation energy than both biodiesel made from it and its blending with kerosene. Pure jatropha oil biodiesel was found to have minimal PM and CO concentrations during the cooking process, with an average magnitude of 209.71 *µ*g/m^3^ and 5.5 mg/kg, respectively. The study indicated that jatropha biodiesel and its blending with kerosene were suitable fuels for existing cookstoves such as wick stoves and lumps with high thermal efficiency. The blending and biodiesel fuels have a higher reaction extent than pure jatropha oil. It may be inferred that pure biodiesel alone could be utilized as a viable liquid fuel in the current wick stoves and lumps, even though the blending of jatropha biodiesel with kerosene has a relatively higher efficiency than biodiesel. With the exception of combining it up to 10% with kerosene, pure jatropha oil was not a suitable fuel for lighting and cooking.

## Figures and Tables

**Table 1:** Characteristics of various fuels.

Property	Jatropha oil	Jatropha biodiesel	Kerosene
Density (kg/m^3^)	910	870	788
Kin. viscosity (mm^2^/s)	30.1	4.5	2.4
Acid value (mgKOH/g)	11	0.35	—
Calorific value (MJ/kg)	38.5	41	43.6
Flashpoint (°C)	211	170	42

**Table 2:** Properties of jatropha oil with kerosene blend.

% Of J. curcas oil (v/v)	% Of kerosene fuel (v/v)	Density (kg/m^3^)	Kinematic viscosity (mm^2^/s)	Calorific value (MJ/kg)
0	100	788	2.4	43.6
5	95	796	2.48	43.4
10	90	800	2.6	43.1
20	80	810	2.85	42.8
30	70	820	3.17	42.4
40	60	830	5.06	42.2
50	50	840	8.21	42.1

**Table 3:** Properties of jatropha oil biodiesel with kerosene blend.

% Of J. curcas biodiesel (v/v)	% Of kerosene fuel (v/v)	Density (kg/m^3^)	Kinematic viscosity (mm^2^/s)	Calorific value (MJ/kg)
0	100		2.4	43.6
5	95	790	2.45	42.42
10	90	795	2.5	42.31
20	80	800	2.58	42.3
30	70	810	2.8	42.28
40	60	820	3.1	42.22
50	50	830	3.4	42.2
60	40	840	3.8	42.18
80	20	850	4.2	42.10
100	0	870	4.5	41

**Table 4:** Mean stove performance indicators.

High power test (cold start)	Units	B0 K100	B5K95	B10K90	B20K80	B30K70	B40K60	B50K50	B60K40	B80K20	B100K0
*Jatropha oil blended with kerosene*											
Time to boil	min	11.6	27	30	—	—	—	—	—	—	—
Fuel consumed	g	22	7.96	4	—	—	—	—	—	—	—
Burning rate	g/min	1.9	0.297	0.13	—	—	—	—	—	—	—
Thermal efficiency	%	60	44.5	10	—	—	—	—	—	—	—
Specific fuel consumption	g/l	25	39.8	42	—	—	—	—	—	—	—
Evaporation rate	g/min	6.9	7.3	1.4	—	—	—	—	—	—	—
Firepower	w	1378	1122	1116							

*Jatropha oil biodiesel blended with kerosene*											
Time to boil	min	11.6	17	19	21	23	25	25.6	25	25.7	26
Fuel consumed	g	22	29	33.4	36	38.9	37.7	39	40	41	43
Burning rate	g/min	1.9	1.7	1.75	1.7	1.69	1.5	1.52	1.6	1.5	1.65
Thermal efficiency	%	60	48	42	39	36.3	37.5	36.2	35.4	34.6	33.89
Specific fuel consumption	g/l	25	30	36	39.4	40	40.3	41.8	42.2	42.5	43
Evaporation rate	g/min	6.8	6.2	5.4	5.51	4.8	5.5	4.7	4.6	5.02	5.6
Firepower	w	1378	1206	1240	1209	1192	1061	1071	1125	1119	1130

*Note.* B: biodiesel or oil, K: kerosene.

**Table 5:** Comparison of different cookstoves in terms of their average performance indicator [11, 29, 30].

Performance indicator	Stove type								
	Traditional 3 stone stove (wood)	Lakech charcoal stove	Tikikil wood stove	Mirt wood stove	Gonze wood stove	Kerosene stoves	Wick jatropha oil-kerosene blend stove	Wick biodiesel stove	Wick biodiesel-kerosene blend stove
Thermal efficiency (%)	10	38	28	48	50	60	10	34	39
Specific fuel consumption (g/l)	929 ^*∗*^	290	198	470 ^*∗*^	617 ^*∗*^	183	41	43	39
Firepower (watts)	—	—	2900	—	—	1300	1119	1130	1153
Emission, CO (mg/kg)	92105	79090	66075	192	186	500	235	5.5	9.4

^*∗*^Gram of wood/kg of food.

## Data Availability

The data that support the findings of this study can be obtained from the corresponding authors on request.

## Nomenclature

*η*_*th*_: Thermal efficiency (%)
*Mn*: Mass of water in the pan (kg)
Cp: Specific heat of water (kJ/kg/°C)
*To*: Starting water temperature (°C)
*Tb*: Boiling water temperature (°C)
*M*_*e*_: Mass of water evaporated (kg)
*L*: Latent heat of evaporation (kJ/kg)
*Mf*: Weight of fuel burnt (kg)
*Hv*: Heating value of the fuel (kJ/kg)
*t*: Time of the test (min)
*CV*: Calorific value of the fuel (J/kg)
*M*1: Mass of water in the pot (g)
*M*2: Mass of water loss through evaporation (g)
*n*: Order of reaction
*k*: Reaction rate constant
*x*: Extent of conversion
Wo: Original weight of sample (g)
*Wt*: Current weight of the sample (g)
*Wf*: Final weight of the sample (g)
*dT*/*dt*: Heating rate symbolized as B
*Ea*: Activation energy
*A*: Frequency factor
*R*: Ideal gas law constant (8.314 J/mol·K)
CO: Carbon monooxide
PM: Particulate matter
*IAP*: Indoor air pollution
WBT: Water-boiling test.

## References

[1] Benti N. E., Gurmesa G. S., Argaw T. (2021). The current status, challenges and prospects of using biomass energy in Ethiopia. *Biotechnology for Biofuels*.

[2] Sovacool B. K. (2012). The political economy of energy poverty: a review of key challenges. *Energy for Sustainable Development*.

[3] Charles Rajesh Kumar J., Majid M. A. (2020). Renewable energy for sustainable development in India: current status, future prospects, challenges, employment, and investment opportunities. *Energy, Sustainability and Society*.

[4] OECD (2018). Achieving clean energy access in sub-Saharan Africa. *Financing Clean Energy Access in Sub-Saharan Africa*.

[5] IEA (2011). Energy for all: financing access for the poor. *World Energy Outlook*.

[6] Guta D. D. (2012). Assessment of biomass fuel resource potential and utilization in Ethiopia: sourcing strategies for renewable energies. *International Journal of Renewable Energy Resources*.

[7] Dinesha P., Kumar S., Rosen M. A. (2019). Performance and emission analysis of a domestic wick stove using biofuel feedstock derived from waste cooking oil and sesame oil. *Renewable Energy*.

[8] Getahun E., Tessema D., Gabbiye N. (2019). *Design and Development of Household Gasifier Cooking Stoves: Natural versus Forced Draft*.

[9] Lam N. L., Smith K. R., Gauthier A., Bates M. N. (2012). Kerosene: a review of household uses and their hazards in low-and middle-income countries. *Journal of Toxicology and Environmental HealthJournal of Toxicology and Environmental Health, Part B Part B: Critical Review*.

[10] Kabeyi M. J. B., Olanrewaju O. A. (2022). Sustainable energy transition for renewable and low carbon grid electricity generation and supply. *Frontiers in Energy Research*.

[11] Adamu L. B., Adem K. D. (2020). Quality and performance evaluation of jatropha oil blended with kerosene for cooking stoves in Ethiopia. *Journal of Renewable Energy*.

[12] Singh R. N. (2011). Straight Vegetable oil: an alternative fuel for cooking, lighting and irrigation pump. *The IIOAB Journal*.

[13] Khalid A., Jaat N., Sapit A. (2015). Performance and emissions characteristics of crude jatropha oil biodiesel blends in a diesel engine. *International Journal of Automotive and Mechanical Engineering*.

[14] Ntalikwa J. W. (2021). Solvent extraction of jatropha oil for biodiesel production: effects of solvent-to-solid ratio, particle size, type of solvent, extraction time, and temperature on oil yield. *Journal of Renewable Energy*.

[15] Aigba P., Anyadiegwu F., Ogoke J. (2021). Characterization of jatropha oil and its biodiesel. *Advances in Environmental Studies*.

[16] Yadav A., Jha P. C. (2013). A case study on biofuel stove technology: jatropha as A biofuel. *International Journal of Technology Enhancements and Emerging Engineering Research*.

[17] Wagutu A. W., Thoruwa T. F. N., Chhabra S. C., Lang’at-Thoruwa C. C., Mahunnah R. L. A. (2010). Performance of a domestic cooking wick stove using fatty acid methyl esters (FAME) from oil plants in Kenya. *Biomass and Bioenergy*.

[18] Natarajan R., Karthikeyan N. S., Agarwaal A., Sathiyanarayanan K. (2008). Use of vegetable oil as fuel to improve the efficiency of cooking stove. *Renewable Energy*.

[19] Lu H., Liu Y., Zhou H., Yang Y., Chen M., Liang B. (2009). Production of biodiesel from Jatropha curcas L. oil. *Computers and Chemical Engineering*.

[20] El-Sayed S. A., Khass T. M., Mostafa M. E. (2023). Thermal degradation behaviour and chemical kinetic characteristics of biomass pyrolysis using TG/DTG/DTA techniques. *Biomass Conversion and Biorefinery*.

[21] Shepherd J. E., Perez F. A. (2008). Kerosene lamps and cookstoves-The hazards of gasoline contamination. *Fire Safety Journal*.

[22] Nagaraju Y. N. Y., Gopal D. L. (2012). Development and performance assessment of a pressurized cook stove using a blend of pongamia oil and kerosene. *International Journal of Scientific Research*.

[23] Leung D. Y. C., Wu X., Leung M. K. H. (2010). A review on biodiesel production using catalyzed transesterification. *Applied Energy*.

[24] Roy M. M., Wang W., Bujold J. (2013). Biodiesel production and comparison of emissions of a DI diesel engine fueled by biodiesel-diesel and canola oil-diesel blends at high idling operations. *Applied Energy*.

[25] Biswas S., Sharma D. K. (2013). Studies on cracking of Jatropha oil. *Journal of Analytical and Applied Pyrolysis*.

[26] Jain S., Sharma M. P. (2012). Application of thermogravimetric analysis for thermal stability of Jatropha curcas biodiesel. *Fuel*.

[27] Li X., Li G., Li J. (2016). Producing petrochemicals from catalytic fast pyrolysis of corn fermentation residual by-products generated from citric acid production. *Renewable Energy*.

[28] Who (2019). *WHO Ambient (Outdoor) Air Quality and Health*.

[29] Dresen E., DeVries B., Herold M., Verchot L., Müller R. (2014). Fuelwood savings and carbon emission reductions by the use of improved cooking stoves in an afromontane forest, Ethiopia. *Land*.

[30] Fedak K. M., Good N., Dahlke J. (2018). Chemical composition and emissions factors for cookstove startup (ignition) materials. *Environmental Science and Technology*.

